# Icariin attenuates titanium-particle inhibition of bone formation by activating the Wnt/β-catenin signaling pathway *in vivo* and *in vitro*

**DOI:** 10.1038/srep23827

**Published:** 2016-03-31

**Authors:** Junhua Wang, Yunxia Tao, Zichuan Ping, Wen Zhang, Xuanyang Hu, Yijun Wang, Liangliang Wang, Jiawei Shi, Xiexing Wu, Huilin Yang, Yaozeng Xu, Dechun Geng

**Affiliations:** 1 Department of Orthopedics, The First Affiliated Hospital of Soochow University, 188, shi zi Road, Suzhou, 215006, China; 2 Orthopedic Institute, Soochow University, 708, ren min Road, Suzhou, 215006, China

## Abstract

Wear-debris-induced periprosthetic osteolysis (PIO) is a common clinical condition following total joint arthroplasty, which can cause implant instability and failure. The host response to wear debris promotes bone resorption and impairs bone formation. We previously demonstrated that icariin suppressed wear-debris-induced osteoclastogenesis and attenuated particle-induced osteolysis *in vivo*. Whether icariin promotes bone formation in a wear-debris-induced osteolytic site remains unclear. Here, we demonstrated that icariin significantly attenuated titanium-particle inhibition of osteogenic differentiation of mesenchymal stem cells (MSCs). Additionally, icariin increased bone mass and decreased bone loss in titanium-particle-induced osteolytic sites. Mechanistically, icariin inhibited decreased β-catenin stability induced by titanium particles *in vivo* and *in vitro*. To confirm icariin mediated its bone-protective effects via the Wnt/β-catenin signaling pathway, we demonstrated that ICG-001, a selective Wnt/β-catenin inhibitor, attenuated the effects of icariin on MSC mineralization *in vitro* and bone formation *in vivo*. Therefore, icariin could induce osteogenic differentiation of MSCs and promote new bone formation at a titanium-particle-induced osteolytic site via activation of the Wnt/β-catenin signaling pathway. These results further support the protective effects of icariin on particle-induced bone loss and provide novel mechanistic insights into the recognized bone-anabolic effects of icariin and an evidence-based rationale for its use in PIO treatment.

Periprosthetic osteolysis (PIO) is a common clinical condition after total joint arthroplasty (TJA), which can lead to implant instability and failure^1^,^2^,^3^. This remains a major orthopedic problem because up to one-third of patients have evidence of osteolysis within 20 years after TJA^4^. To date, the only established treatment for implant failure is revision surgery, which has a higher cost, a poorer clinical outcome, and a shorter survival duration when compared with the primary TJA. Although the precise mechanism of PIO remains unclear, it is generally believed that it is primarily caused by a biological reaction to wear debris accumulated at the bone—implant interface leading to local bone loss due to an imbalance between osteoblastic bone formation and osteoclastic bone sorption^2^,^5^.

Icariin, the main active flavonol glucoside in Epimedium, has demonstrated bone-protective actions. In postmenopausal women, icariin exerted beneficial effects in preventing bone loss^6^. Nian *et al.* recently reported that icariin treatment stimulated bone formation and increased bone mass^7^. *In vitro* studies revealed that the increased bone mass was associated with the differentiation of bone marrow stromal cells and enhanced expression of various proteins critical to bone matrix deposition^8^,^9^,^10^,^11^. In addition, icariin inhibited the formation and activation of osteoclasts^12^,^13^. These results suggest that icariin prevents bone loss by stimulating bone formation and suppressing bone resorption. We previously demonstrated that icariin suppressed wear-debris-induced osteoclastogenesis^14^ and attenuated particle-induced osteolysis *in vivo*^15^. However, whether icariin could promote bone formation in an osteolytic site induced by wear debris remains unclear.

Osteoblasts are derived from mesenchymal stem cells (MSCs) and are bone-forming cells. The lineage is tightly regulated by many bone-forming signals, particularly the Wnt/β-catenin signaling pathway^16^,^17^,^18^. Activation of Wnt/β-catenin is essential for proper bone development, and inhibition of this signaling pathway down-regulates bone formation^18^. We recently demonstrated in a murine calvarial model of osteolysis that activation of the β-catenin signaling pathway significantly reduced bone resorption and promoted bone formation at an osteolytic site induced with wear debris^19^. In addition, activation of the Wnt/β-catenin signaling pathway was critical in icariin-related bone-protective effects^20^. Therefore, we hypothesized that icariin treatment would promote bone formation in an osteolytic site induced by wear debris via the Wnt/β-catenin signaling pathway.

In the present study, we provide evidence that icariin attenuated titanium (Ti)-particle inhibition of osteoblast differentiation through stabilization of β-catenin protein and activation of the Wnt/β-catenin signaling pathway. The results of this study provide a possible mechanistic explanation for the protective effect of icariin against wear-debris-induced bone loss and provide a rationale for icariin use in the treatment of PIO.

## Results

### Icariin attenuates Ti-particle inhibition of osteogenic differentiation in MSCs

**Figure 1:**
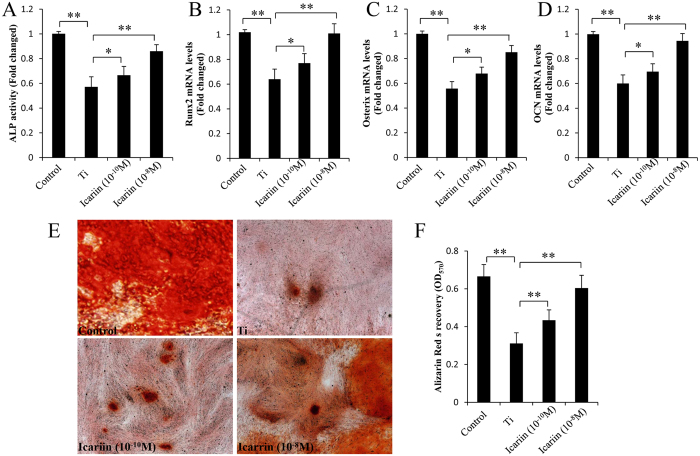
Icariin attenuates Ti particles inhibition of osteogenic differentiation of mMSCs. mMSCs were pretreated with 10^−10^ M or 10^−8^ M icariin and subsequently induced to differentiate in the presence of Ti particles for 3 days. Icariin maintained ALP activity (**A**), Runx2 (**B**) and Osterix (**C**) gene expression in a dose dependent manner. (**D**) Icariin maintained OCN expression. Cell differentiation was induced for 10 days. (**E**,**F**) Icariin attenuates Ti particle inhibition of mineralization in MSCs. Cell differentiation was induced for 21 days. Data are presented as mean ± SD, *p < 0.05, **p < 0.01, one-way ANOVA and Tukey post-hoc pairwise comparisons.

To investigate whether Ti particles affected MSC differentiation, the cells were grown in osteogenic differentiation-inducing media and stimulated with/without Ti particles. Ti particles significantly inhibited ALP activity (Fig. 1A), an early differentiation marker. In addition, RT-PCR results demonstrated that Ti particles significantly reduced the mRNA levels of Runx2 and Osterix (Fig. 1B,C), two master osteoblast-specific transcription factors. Consistent with this, Ti particles also reduced OCN expression (Fig. 1D). At the end of this study, MSCs were stained using ARS. Ti particles significantly decreased the staining density of ARS in visible observation quantification by spectrophotometry when compared with the control group (Fig. 1E,F). We also examined the effect of icariin on Ti-particle-induced inhibition on MSC differentiation. Although Ti particles inhibited osteogenic differentiation mediated by MSCs, this was potently attenuated by icariin in a dose-dependent manner (Fig. 1).

### Icariin and the Wnt/β-catenin signaling pathway

**Figure 2:**
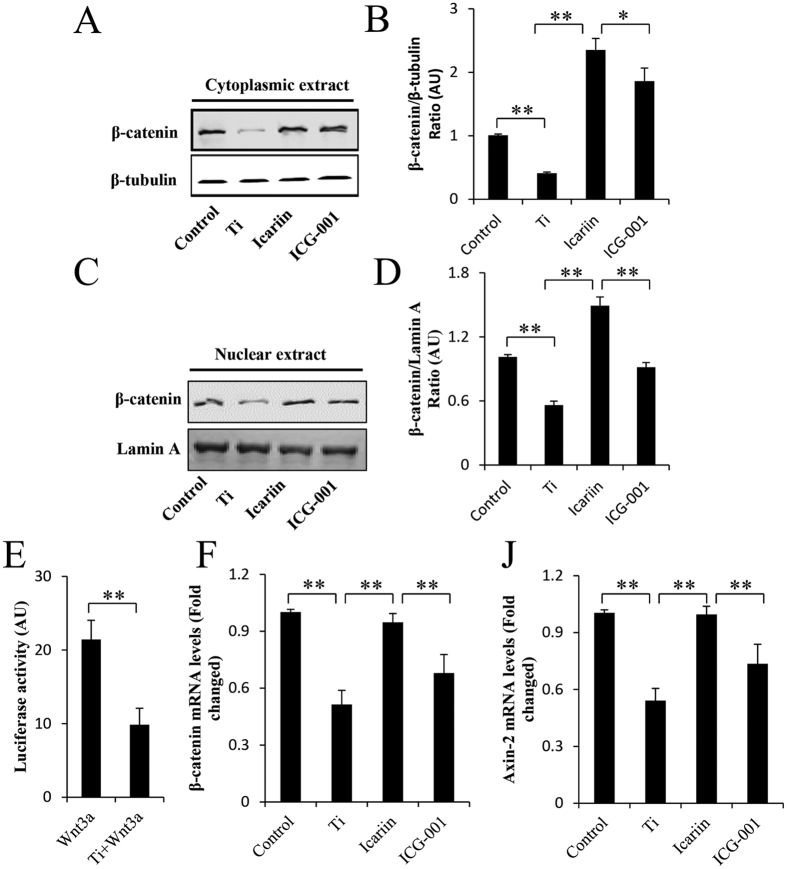
Icariin restore the stability of β-catenin inhibited by Ti particles. (**A**–**D**) Immunoblot of mMSCs show the cytoplasm and nucleus levels of β-catenin after stimulation of Ti particles with or without icariin or ICG-001. (**E**) Luciferase activities were measured in cell lysates. (**F**,**J**) The expression of β-catenin and axin-2 were determined by real-time RT-PCR. Data are presented as mean ± SD, *p < 0.05, **p < 0.01, one-way ANOVA and Tukey post-hoc pairwise comparisons.

To understand the molecular mechanisms by which icariin attenuates Ti–particle inhibition of osteogenic differentiation of MSCs, we screened several signaling pathways and key molecules associated with MSC differentiation. The Wnt/β-catenin signaling pathway plays an essential role in the regulation of bone formation. Interestingly, we found that Ti-particle stimulation reduced the cytosolic and nuclear levels of β-catenin in mMSCs (Fig. 2A–D). In addition, we found that Ti particles impaired β-catenin-dependent transcription induced by Wnt-3a as determined by the Topflash reporter assay (Fig. 2E). To determine whether Ti-particle-induced inhibition of Wnt/β-catenin activity could be reversed by icariin, MSCs were pretreated with 10^−8^ M icariin followed by the application of Ti particles. Western blot analysis revealed that icariin inhibited β-catenin degradation induced by Ti particles. RT-PCR results showed that the mRNA levels of β-catenin and the β-catenin target gene axin-2 were significantly increased in the icariin-treated group when compared with that in the untreated group (Fig. 2F,J). These results demonstrated that icariin attenuated Ti-particle inhibition of the Wnt/β-catenin signaling pathway in MSCs.

**Figure 3:**
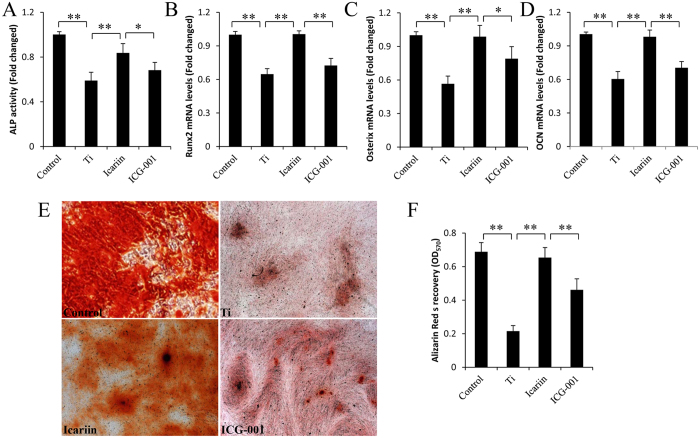
ICG-001 reversed icariin effects on osteogenic differentiation of mMSCs. (**A**) ALP activity, (**B**) Runx2 and (**C**) Osterix mRNA levels were measured after 3 days of incubation. (**D**) OCN levels were determined at 10 days. (**E**,**F**) mMSCs mineralization assessed by ARS after culture for 21 days. Extraction and colorimetric quantification of ARS confirms ICG-001 substantially attenuates mineralization of MSCs even in the presence of icariin. Data are presented as mean ± SD, *p < 0.05, **p < 0.01, one-way ANOVA and Tukey post-hoc pairwise comparisons.

We also investigated whether the protective effects of icariin were mediated via the β-catenin signaling pathway. MSCs were pretreated with the Wnt/β-catenin inhibitor ICG-001 (10 μM) and then stimulated with icariin and Ti particles for the indicated time. ICG-001 attenuated icariin-mediated protective effects on β-catenin activity (Fig. 2). Consistent with this, ICG-001 also inhibited osteogenic differentiation of MSCs. ICG-001 treatment abolished icariin-mediated promotion of ALP activity and Runx2, Osterix, and OCN gene expression. Moreover, we found that inhibition of β-catenin activity also attenuated icariin-induced osteogenic mineralization mediated by MSCs (Fig. 3). These observations strongly suggest that icariin-attenuated Ti-particle inhibition of MSC differentiation is at least in part via the activation of the Wnt/β-catenin signaling pathway.

### Icariin promotes bone formation and inhibits bone resorption in the osteolytic site induced by Ti particles

**Figure 4:**
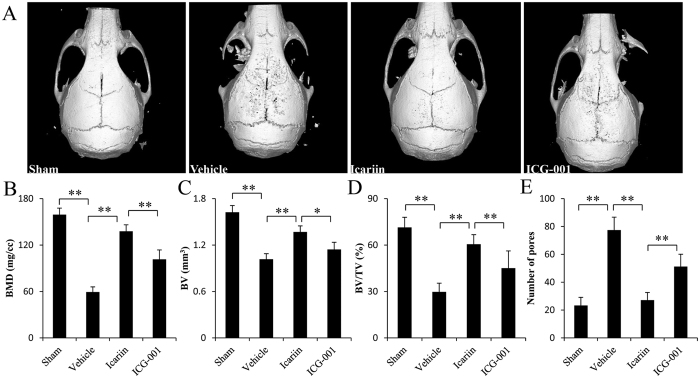
Icariin increases bone mass and prevents bone loss in murine calvariae model. (**A**) micro-CT reconstruction, (**B**) BMD, (**C**) BV, (**D**) BV/TV, and (**E**) number of pores of each sample within the ROI were measured. n = 7 per groups. Data are presented as mean ± SD, *p < 0.05, **p < 0.01, one-way ANOVA and Tukey post-hoc pairwise comparisons.

**Figure 5:**
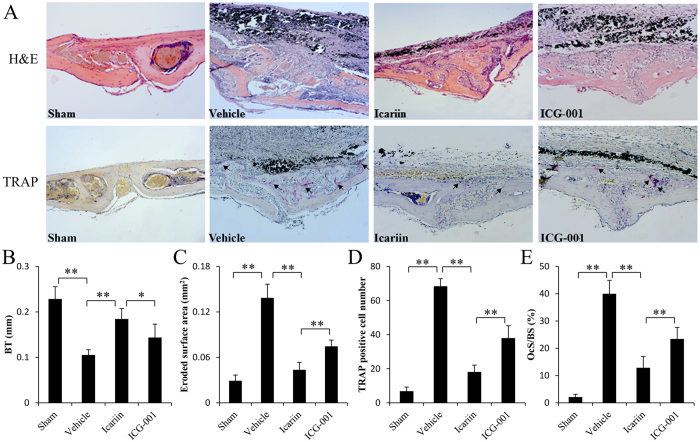
Histological staining and histomorphometric analysis of calvaria sections. (**A**) Hematoxylin and eosin (**H&E**) and tartrate-resistant acid phosphatase (TRAP)-stained histological slices. (**B**) Histomorphometric analysis of the bone thickness, (**C**) the eroded surface area, (**D**) the number of TRAP-positive multinucleated osteoclasts and (**E**) the percentage of osteoclast surface per bone surface (OcS/BS, %) within the ROI in each group were measured. n = 7 per groups. Data are presented as mean ± SD, *p < 0.05, **p < 0.01, one-way ANOVA and Tukey post-hoc pairwise comparisons.

**Figure 6:**
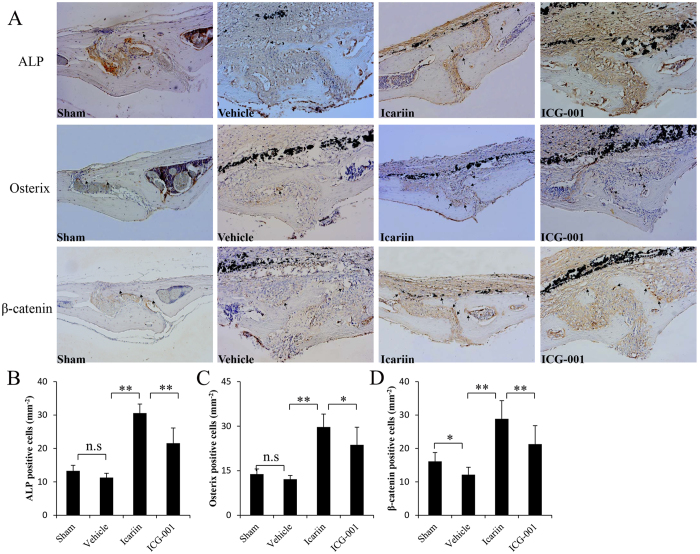
Expression of ALP, Osterix and β-catenin in the calvariae of mice. (**A**) Representative Immunohistochemical images of ALP, Osterix and β-catenin (brown, indicated by the arrows). Semi-quantitative analysis showed that icariin treatment obviously increased ALP (**B**), Osterix (**C**) and β-catenin (**D**) positive cells in the calvariae of mice stimulated with Ti particles, while ICG-001 treatment attenuates the effects of icariin. n = 7 per groups. Data are presented as mean ± SD, *p < 0.05, **p < 0.01, one-way ANOVA and Tukey post-hoc pairwise comparisons.

To investigate whether icariin promotes bone formation *in vivo*, the murine calvarial model was used to mimic the molecular pathogenesis of PIO. μCT analysis revealed that the BMD of icariin-treated mice was significantly higher compared with that of Ti-particle-stimulated mice (Fig. 4A,B). Similarly, the BV and BV/TV were significantly higher in icariin-treated mice compared with Ti-particle-stimulated mice (Fig. 4C,D), consistent with the higher bone thickness shown in H&E staining and bone histomorphometry analysis results (Fig. 5A,B). To determine whether the increased BMD was due to increased osteoblast function, immunohistological staining was applied to detect ALP and Osterix expression. In vehicle mice exposed to Ti particles alone, an almost complete absence of ALP- and Osterix-positive cells was observed. Icariin treatment reversed this effect, with increased ALP- and Osterix-positive cells in the ROI of the calvariae (Fig. 6).

Because osteoclastogenesis and bone resorption are enhanced in PIO, we investigated the effect of icariin on accelerated bone resorption in a mouse calvarial model. μCT analysis showed increased pitting and porosity in Ti-particle-stimulated mice (Fig. 4A,E). In contrast, icariin significantly decreased bone resorption. H&E staining clearly revealed resorption in sections obtained from the vehicle group. TRAP staining demonstrated that multiple TRAP-positive cells were present along the eroded bone surface in the vehicle group (Fig. 5A). Histomorphometric results demonstrated that icariin treatment significantly reduced the area of eroded surface, the number of TRAP-positive cells, and OCs/BS induced by Ti particles (Fig. 5C–E).

Previous studies have demonstrated the inhibition of the Wnt/β-catenin signaling pathway in osteolytic disease^17^,^19^. Therefore, we investigated whether icariin activates the Wnt/β-catenin signaling pathway in an osteolytic site induced by Ti particles. As predicted, immunohistochemical analysis of sections obtained from the icariin group showed greater positive staining for β-catenin, whereas fewer β-catenin-positive cells were observed in Ti-particle-stimulated mice. Moreover, the number of β-catenin-positive cells was significantly decreased when ICG-001, a Wnt/β-catenin inhibitor, was administrated to icariin-treated mice, further indicating that icariin activates the Wnt/β-catenin signaling pathway in an osteolytic scenario (Fig. 6). In addition, local treatment with ICG-001 attenuated the effects of icariin on bone mass and on ALP and osterix expression, which suggests that icariin promotes new bone formation via the Wnt/β-catenin signaling pathway in an osteolytic site induced by Ti particles.

## Discussion

Icariin, a major active component isolated from plants in the Epimedium family, has been used in the treatment of bone fractures and osteoporosis in traditional Chinese medicine. We previously demonstrated that icariin treatment significantly decreased Ti-particle-induced bone resorption *in vivo* and inhibited particle-induced osteoclastogenesis in a dose-dependent manner *in vitro*^14^,^15^, which indicates that icariin might be a candidate for the treatment of PIO. However, osteoclast-function targeting agents, including nitrogen-containing bisphosphonates, have been unsuccessful in treating PIO^21^,^22^, and several authors proposed that even when osteoclast activity was reduced, no osteoblastic repair occurred and the lytic bone failed to heal^22^,^23^. Therefore, it is necessary to investigate whether there is any compensatory osteoblastic or anabolic response during the icariin treatment process.

The results of the present study demonstrated that icariin treatment significantly reduced wear-debris-induced inhibition of osteogenic differentiation in mMSCs and promoted osteoblastic bone formation in the osteolytic site in a murine calvarial model. Additional experiments indicated that this effect was mediated by activation of the Wnt/β-catenin signaling pathway. The bone resorption model was introduced by Merkel *et al.* and is a widely used particle-based model of wear-debris-induced osteolysis^24^. Ti particles, which are frequently generated from many different orthopedic prostheses, were used in this study to mimic debris released during aseptic loosening. The data indicate that Ti-particle stimulation significantly decreased osteoblast differentiation and osteoblastic bone formation, and that this decrease was mitigated by icariin treatment.

The osteoblast is the main cell in the formation of bone tissue that constitutes the skeletal system, and that participates in the processes that influence the stability of the bone at the margin of the bone implant^2^,^5^. The host response of osteoblasts and their precursors to wear debris is critical to periprosthetic bone formation^5^. Growing evidence suggests that wear debris, including Ti, polymeric and polymethyl methacrylate, impairs the function of mature osteoblasts as well as inhibiting bone formation and differentiation of osteoblast precursors^23^,^24^,^25^,^26^,^27^,^28^,^29^,^30^, which is consistent with the current study. In addition, several authors have demonstrated that bone formation markers are decreased in patients with aseptic loosening^31^,^32^. Although the underlining mechanisms of wear-debris-induced inhibition of bone formation remain unclear, the regulation of bone formation clearly plays a dominant role in the pathogenesis of PIO and is an important therapeutic target for the treatment of this destructive bone disease.

Icariin treatment attenuated Ti-particle-induced inhibition of osteogenic activity in the current study. Recently, the adverse effects of wear debris on the proliferation, differentiation, and osteogenic functions of osteoprogenitor cells have been identified^5^,^27^,^29^. Here, we also observed a lower expression of ALP, OCN, Runx2, and Osterix in osteoprogenitor cells stimulated with Ti particles. Interestingly, icariin treatment significantly diminished the adverse effects of Ti particles on osteogenic activity, which is consistent with previous results that icariin treatment increased osteoblast differentiation of MSCs^8^,^9^. In addition, the morphometric analysis revealed higher BV, BV/TV, BMD, and bone thickness in the icariin-treated animals compared with those in the untreated mice; further supporting the concept that icariin exerts bone-protective actions. Moreover, icariin treatment increased Osterix expression *in vitro* and *in vivo*. The important role of Osterix in MSC differentiation suggests that the effect of icariin on bone may occur by enhancing osteogenic differentiation of MSCs. These results from the current and related studies strongly suggest that osteoblasts and osteoprogenitors are indeed the target of icariin, and icariin treatment promotes bone formation in an osteolysis scenario stimulated using Ti particles.

The Wnt/β-catenin signaling pathway, which is regulated by ubiquitin-mediated proteasomal degradation of β-catenin^17^, plays a key role in osteoblast differentiation and bone formation, and there is increasing data to suggest a role for this pathway in the development of osteolytic disease, including PIO^19^,^33^. In support of this, we demonstrated a clear decrease in β-catenin expression in Ti-particle-stimulated osteoprogenitor cells and in a murine calvarial osteolysis model, which is closely associated with Wnt signaling activity. In addition, icariin treatment significantly increased the levels of β-catenin *in vivo* and *in vitro*, and administration of β-catenin inhibitor reversed these effects. These results suggest that icariin attenuates Ti-particle-induced inhibition of bone formation by activation of the Wnt/β-catenin signaling pathway in osteoprogenitor cells.

However, there are several limitations to the current study. First, to mimic the osteolysis scenario, commercial Ti particles were used rather than polyethylene particles, which are the main cause of PIO^29^,^30^,^34^,^35^. However, it has been demonstrated that these Ti particles induced osteolysis in bone tissue around the prosthetic implant by mechanisms similar to polyethylene particles^30^,^36^. Moreover, polyethylene particles are difficult to use in cell culture because these particles tend to float away from the cells. Second, it is important to examine the same ROI in all subjects to minimize variability. Therefore, the size of the ROI was kept constant and anatomical landmarks of the coronal and sagittal sutures were used to standardize the analysis. Third, the murine calvarial model was used in the current study. To generate osteolysis, a fixed amount of Ti particles was implanted rather than being continuously generated by wear as in implant patients, a difference described by von Knoch *et al.*^36^. Therefore, a larger animal model with continuous particle generation would provide a better animal model for future studies.

In conclusion, this study clearly showed that icariin can induce new bone formation and prevent bone loss at an osteolytic site caused by Ti-particle stimulation. These effects may be mediated by activation of the Wnt/β-catenin signaling pathway and increasing the osteogenic activity of MSCs. These findings further support the protective effects of icariin on particle-induced bone loss and provide novel mechanistic insights into the recognized bone-anabolic effects of icariin and an evidence-based rationale for its use in the treatment of PIO.

## Materials and Methods

### Ti-particle preparation

Commercially pure Ti particles were purchased from Johnson Matthey Company (Ward Hill, MA, USA). The mean particle diameter was 3.32 ± 2.39 μm, with 90% < 3.6 μm. Ti particles were sterilized by baking at 180 °C for 6 h, and then treatment twice in 70% ethanol for 24 h at room temperature. After washing three times in sterile phosphate-buffered saline (PBS), the particles were dried under a sterile bench. Only endotoxin-free particles, as determined by means of the Limulus Amoebocyte Lysate assay (Biowhittaker, Walkersville, MD, USA), were used in the current study.

### Cell cultures

Mouse MSCs (mMSCs; Cyagen, Guangzhou, China) were cultured in MSC basal media, including MSC growth supplement (MCGS), L-glutamine, penicillin, and streptomycin at 37 °C and 5% CO_2_. For differentiation experiments, cells were cultured with mMSC Differentiation Basal Medium-osteogenic containing β-glycerophosphate, dexamethasone, ascorbic acid, MCGS, L-glutamine, penicillin, and streptomycin. After confluence, the cells were treated with 10^−10^ M or 10^−8^ M icariin (Sigma, St. Louis, MO, USA) with/without 10 μM Wnt/β-catenin inhibitor ICG-001 (Tocris Bioscience, Bristol, UK), starting 4 h before treatment with 0.1 mg/mL Ti particles. The concentration of Ti particles used for incubation was similar to that of wear debris retrieved from periprosthetic tissues^36^. In addition, 10^−10^ M and 10^−8^ M icariin significantly enhanced osteoblast differentiation without cytotoxicity as previously described^10^.

### Alkaline phosphatase (ALP) activity

ALP activity was determined as a marker of osteoblast differentiation in response to Ti particles and icariin with/without ICG-001. Briefly, mMSC cells were seeded at a density of 9 × 10^4^ cells/well in 24-well plates. On confluence, the medium was replaced with fresh medium containing Ti particles with/without icariin and with/without ICG-001, and incubated for 3 days. ALP activity was determined at 405 nm using p-nitrophenyl phosphate (Sigma) as the substrate and the total protein content was determined using a BCA protein kit (Pierce Chemical Company, Rockford, IL, USA) as previously described^33^.

### Alizarin red S (ARS) staining

The mineralization of mMSCs, which were differentiation-induced for 21 days, was determined by ARS staining. The cells were rinsed twice using PBS, fixed in 4% paraformaldehyde at 4 °C for 30 min, and washed using deionized water. Cell staining was performed using 40 mM ARS solution (pH 4.2; Sigma) for 30 min at room temperature. Subsequently, the cells were rinsed three times with double-distilled water and washed using PBS for 15 min to reduce nonspecific ARS staining. The stained cells were observed under a microscope. To quantify the mineralization, the stained layers were solubilized by the addition of 10% cetylpyridinium chloride (pH 7.0; Sigma) as previously described^11^. Dye absorbance was determined at 570 nm. Six wells were analyzed per experiment and the experiments performed in triplicate.

### Luciferase assay

Semi-confluent mMSCs were transfected using 2-μg Wnt reporter constructs containing wild-type (Topflash) or mutated (Fopflash) LEF/TCF-binding sites using Genefectine transfection reagent (Sigma), following the manufacturer’s protocol. Cells were co-transfected with 0.2 μg β-galactosidase reporter vector as a control for transfection efficiency. After transfection, the cells were treated with 20 ng/mL Wnt-3a (R&D Systems, Minneapolis, MN, USA), starting 1 h before Ti-particle treatment for 24 h. The luciferase activity was determined using a Dual-Luciferase Assay kit (Promega, Sunnyvale, CA, USA) as previously described^33^.

### RNA extraction and real-time quantitative reverse transcription polymerase chain reaction (qRT-PCR)

Total RNA were isolated as described previously using TRIzol reagent and reverse transcribed^34^,^37^. RNA integrity was assessed by light absorbance at 260 and 280 nm and by agarose gel electrophoresis with ethidium bromide staining. Real-time RT-PCR was performed using an SYBR Premix Ex Taq kit (TaKaRa Biotechnology, Otsu, Japan) and a TAKARA TP800 PCR Thermal Cycler Dice Detection system at 95 °C for 10 min for initial denaturation, followed by 40 cycles of 95 °C for 15 s, 60 °C for 30 s, and 72 °C for 30 s. All reactions were performed in triplicate and analyzed by the 2-ΔΔCT method^38^. Glyceraldehyde 3-phosphate dehydrogenase (GAPDH) served as an internal control. The gene-specific primers for osteocalcin (OCN), Runx2, Osterix, β-catenin, axin-2, and GAPDH were as follows: OCN forward 5′-TCCCACACAGCAGCTTGGCCC-3′ and reverse 5′-TGAGGCTCCAAGGTAGCGCCG-3′; Runx2 forward 5′-TTGACCTTTGTCCCAATGC-3′ and reverse 5′-AGGTTGGAGGCACACATAGG-3′; Osterix forward 5′-TGAGCTGGAACGTCACGTGC-3′ and reverse 5′-AAGAGGAGGCCAGCCAGACA-3′; β-catenin forward 5′-ACGGTGCCGCGCCGCTTATA-3′ and reverse 5′-TAGCCATTGTCCACGCAGCGG-3′; axin-2 forward 5′-GTCTCTACCTCATTTCCCGAGAAC-3′ and reverse 5′-CGAGATCAGCTCAGCTGCAA-3′; GAPDH forward 5′-GAGAAGGCTGGGGCTCATTT-3′ and reverse 5′-CCAATATGATTCCACCCATG-3′.

### Protein isolation and western blot analysis

Nuclear and cytoplasmic extracts were obtained using a nuclear extraction kit (Sigma). Protein quantification was performed using the BCA protein assay reagent (Pierce). Twenty micrograms of each sample were run under sodium dodecyl sulfate polyacrylamide gel electrophoresis and transferred to a polyvinylidene fluoride membrane, which was blocked and probed using primary antibody against β-catenin (Cell Signaling Technology, Cambridge, MA, USA) overnight at 4 °C. Subsequently, blots were washed using Tris-buffered saline with Tween 20 (10 mM Tris-HCl, 50 mM NaCl, 0.25% Tween 20) and incubated with horseradish-peroxidase-conjugated secondary antibody (Cell Signaling Technology). The protein was detected using enhanced chemiluminescence reagents. As a loading control, anti-β-tubulin and anti-Lamin A (Cell Signaling Technology) antibodies were used.

### Mouse calvarial model

For the *in vivo* study, we used a Ti-particle-induced mouse calvarial model. The animal studies were performed in accordance with the principles and procedures of the National Institutes of Health (NIH) Guide for the Care and Use of Laboratory Animals and the guidelines for animal treatment of the First Affiliated Hospital of Soochow University. All experiments were approved by the Ethics Committee of the First Affiliated Hospital of Soochow University. Briefly, 28 female 6–7-week-old C57BL/6 mice were assigned randomly to four groups: PBS control (sham); Ti particles in PBS group (vehicle); Ti particles and icariin (icariin group); and Ti particles, icariin and ICG-001 (ICG group). The mice were anesthetized using an intraperitoneal injection of 50 mg kg^−1^ pentobarbital. Either no Ti particles (sham) or 20 mg of Ti particles (vehicle, icariin, and ICG groups) were placed directly on the surface of the calvarial bone. Mice in the icariin and ICG groups were gavage-fed with icariin at 0.3 mg g^−1^ day^−1^. In addition, the icariin-treated mice received a 20-μL injection of PBS or ICG-001 (10 μg) at the surgery site prior to particle application and then daily until sacrifice. Mice in the sham and vehicle groups received PBS daily. Before surgery, all mice received a subcutaneous injection of carprofen (4 mg kg^−1^; KDN PHARM, Qingdao, China), and the oral antibiotic enrofloxacin (100 mg mL^−1^; GuideChem, Nanjing, China) was administered in the drinking water for 3 days after the operation. No adverse effects or mortality occurred during the experiment. The calvariae were collected from the mice 2 weeks after the operation and dissected for molecular, micro-computed tomography (μCT), and histological analyses.

### μCT scanning

The fixed calvaria were analyzed by μCT using a SkyScan1176 scanner and associated analysis software (SkyScan, Aartselaar, Belgium). The scanning protocol was set as an isometric resolution of 18 μm and the X-ray energy settings were 80 kV and 100 μA. Three-dimensional image reconstructions were obtained using the manufacturer’s software. As previously described, a cylindrical region of interest (ROI; 3 × 3 × 1 mm), with the midline suture at its center, was selected for quantitative analysis of the particle-induced osteolysis^35^,^39^. The bone mineral density (BMD, mg/cc), bone volume (BV, mm^3^), BV to tissue volume ratio (BV/TV, %), and number of pores of each sample were obtained using CT Analyser software (Skyscan) as previously described^35^,^40^,^41^.

### Histological and immunohistochemical analyses

After μCT scanning, the calvaria were decalcified and paraffin embedded using standard procedures. Five-micrometer-thick paraffin-embedded calvarial bone sections were cut in the coronal plane using a microtome. Sections were prepared for tartrate-resistant acid phosphatase (TRAP) and hematoxylin and eosin (H&E) staining. The stained sections were observed under a high-quality light microscope at a magnification of ×20. The ROI was defined as previously recommended^35^. Histomorphometric analysis was performed using Image Pro Plus software 6.0 (Media Cybernetics, Silver Spring, MD, USA). The eroded surface area (mm^2^), bone thickness (mm), number of TRAP-positive cells, and osteoclast surface per bone surface (OcS/BS, %) were determined as described previously^35^,^39^,^40^,^41^.

For immunohistological analysis of β-catenin, ALP, and Osterix, sections were incubated with the respective primary antibody (all Abcam, Shanghai, China) overnight at 4 °C. After washing, the sections were incubated with a biotin-conjugated secondary antibody for 30 min, rinsed, and incubated with avidin-biotin enzyme reagent for 30 min at 37 °C. Color was developed using 3,3′-diaminobezidine tetrahydrochloride and hematoxylin as a counterstain. The positive cells were counted using a microscope by two independent observers.

### Statistical analysis

The data are expressed as means ± standard deviation (SD). All analyses were performed using the SPSS 11.0 software (SPSS, Chicago, IL, USA). The results were first assessed using a Kolmogorov-Smirnov test to ensure normality and homogeneity of variance. Statistical analyses were performed using a one-way analysis of variance with Tukey post-hoc pairwise comparisons. A value of *p* < 0.05 indicated a significant difference between groups.

## Additional Information

**How to cite this article**: Wang, J. *et al.* Icariin attenuates titanium-particle inhibition of bone formation by activating the Wnt/β-catenin signaling pathway *in vivo* and *in vitro*. *Sci. Rep.* **6**, 23827; doi: 10.1038/srep23827 (2016).

## References

[b1] Wooley P. H. & Schwarz E. M. Aseptic loosening. Gene Ther. 11, 402–7 (2004). 14724679 10.1038/sj.gt.3302202

[b2] Holt G., Murnaghan C., Reilly J. & Meek R. M. The biology of aseptic osteolysis. Clin. Orthop. Relat. Res. 460, 240–52 (2007). 17620815 10.1097/BLO.0b013e31804b4147

[b3] Urban R. M. *et al.* Successful long-term fixation and progression of osteolysis associated with first-generation cementless acetabular components retrieved post mortem. J. Bone Joint Surg. Am. 94, 1877–85 (2012). 23079880 10.2106/JBJS.J.01507 PMC3489071

[b4] Keener J. D. *et al.* Twenty-five-year results after Charnley total hip arthroplasty in patients less than fifty years old: a concise follow-up of a previous report. J. Bone Joint Surg. Am. 85, 1066–72 (2003). 12784004 10.2106/00004623-200306000-00013

[b5] Goodman S. B., Ma T., Chiu R., Ramachandran R. & Smith R. L. Effects of orthopaedic wear particles on osteoprogenitor cells. Biomaterials. 27, 6096–101 (2006). 16949151 10.1016/j.biomaterials.2006.08.023

[b6] Zhang G., Qin L. & Shi Y. Epimedium-derived phytoestrogen flavonoids exert beneficial effect on preventing bone loss in late postmenopausal women: a 24-month randomized, double-blind and placebo-controlled trial. J. Bone Miner. Res. 22, 1072–9 (2007). 17419678 10.1359/jbmr.070405

[b7] Nian H., Ma M. H., Nian S. S. & Xu L. L. Antiosteoporotic activity of icariin in ovariectomized rats. Phytomedicine. 16, 320–6 (2009). 19269147 10.1016/j.phymed.2008.12.006

[b8] Zhai Y. K. *et al.* Icariin stimulates the osteogenic differentiation of rat bone marrow stromal cells via activating the PI3K-AKT-eNOS-NO-cGMP-PKG. Bone. 66, 189–98 (2014). 24956021 10.1016/j.bone.2014.06.016

[b9] Ming L. G., Chen K. M. & Xian C. J. Functions and action mechanisms of flavonoids genistein and icariin in regulating bone remodeling. J. Cell Physiol. 228, 513–21 (2013). 22777826 10.1002/jcp.24158

[b10] Hsieh T. P., Sheu S. Y., Sun J. S., Chen M. H. & Liu M. H. Icariin isolated from Epimedium pubescens regulates osteoblasts anabolism through BMP-2, SMAD4, and Cbfa1 expression. Phytomedicine. 17, 414–23 (2010). 19747809 10.1016/j.phymed.2009.08.007

[b11] Song L., Zhao J., Zhang X., Li H. & Zhou Y. Icariin induces osteoblast proliferation, differentiation and mineralization through estrogen receptor-mediated ERK and JNK signal activation. Eur. J. Pharmacol. 714, 15–22 (2013). 23764463 10.1016/j.ejphar.2013.05.039

[b12] Hsieh T. P., Sheu S. Y., Sun J. S. & Chen M. H. Icariin inhibits osteoclast differentiation and bone resorption by suppression of MAPKs/NF-kappaB regulated HIF-1alpha and PGE(2) synthesis. Phytomedicine. 18, 176–85 (2011). 20554188 10.1016/j.phymed.2010.04.003

[b13] Chen K. M. *et al.* Icariin inhibits the osteoclast formation induced by RANKL and macrophage-colony stimulating factor in mouse bone marrow culture. Pharmazie. 62, 388–91 (2007). 17557750

[b14] Cui J. *et al.* Inhibitory effect of icariin on Ti-induced inflammatory osteoclastogenesis. J. Surg. Res. 192, 447–53 (2014). 24969548 10.1016/j.jss.2014.05.038

[b15] Shao H. *et al.* Icariin protects against titanium particle-induced osteolysis and inflammatory response in a mouse calvarial model. Biomaterials. 60, 92–9 (2015). 25985156 10.1016/j.biomaterials.2015.04.048

[b16] Zhong Z. *et al.* Wntless functions in mature osteoblasts to regulate bone mass. Proc. Natl. Acad. Sci. USA 109, E2197–204 (2012). 22745162 10.1073/pnas.1120407109 PMC3421196

[b17] Clevers H. & Nusse R. Wnt/beta-catenin signaling and disease. Cell. 149, 1192–205 (2012). 22682243 10.1016/j.cell.2012.05.012

[b18] Lin C. *et al.* Sclerostin mediates bone response to mechanical unloading through antagonizing Wnt/beta-catenin signaling. J. Bone Miner. Res. 24, 1651–61 (2009). 19419300 10.1359/jbmr.090411

[b19] Geng D. *et al.* Pharmaceutical inhibition of glycogen synthetase kinase 3 beta suppresses wear debris-induced osteolysis. Biomaterials. 69, 12–21 (2015). 26275858 10.1016/j.biomaterials.2015.07.061

[b20] Li X. F. *et al.* Icariin Augments Bone Formation and Reverses the Phenotypes of Osteoprotegerin-Deficient Mice through the Activation of Wnt/beta -Catenin-BMP Signaling. Evid. Based Complement Alternat. Med. 2013, 652317 (2013). 24348713 10.1155/2013/652317 PMC3835354

[b21] Schwarz E. M. What potential biologic treatments are available for osteolysis? J. Am. Acad. Orthop. Surg. 16 Suppl 1, S72–5 (2008). 18612019 10.5435/00124635-200800001-00015

[b22] Saleh K. J., Thongtrangan I. & Schwarz E. M. Osteolysis: medical and surgical approaches. Clin. Orthop. Relat. Res. 427, 138–47 (2004). 15552150

[b23] Tsutsumi R. *et al.* Differential effects of biologic versus bisphosphonate inhibition of wear debris-induced osteolysis assessed by longitudinal micro-CT. J. Orthop. Res. 26, 1340–6 (2008). 18404739 10.1002/jor.20620 PMC2742224

[b24] Merkel K. D. *et al.* Tumor necrosis factor-alpha mediates orthopedic implant osteolysis. Am. J. Pathol. 154, 203–10 (1999). 9916934 10.1016/s0002-9440(10)65266-2 PMC1853441

[b25] Qin A. *et al.* Prevention of wear particle-induced osteolysis by a novel V-ATPase inhibitor saliphenylhalamide through inhibition of osteoclast bone resorption. PLoS One. 7, e34132 (2012). 22509274 10.1371/journal.pone.0034132 PMC3324493

[b26] Liu X. *et al.* Strontium ranelate inhibits titanium-particle-induced osteolysis by restraining inflammatory osteoclastogenesis *in vivo*. Acta. Biomater. 10, 4912–8 (2014). 25078426 10.1016/j.actbio.2014.07.025

[b27] Choi M. G. *et al.* Effects of titanium particle size on osteoblast functions *in vitro* and *in vivo*. Proc. Natl. Acad. Sci. USA 102, 4578–83 (2005). 15755807 10.1073/pnas.0500693102 PMC555523

[b28] Cordova L. A. *et al.* Inhibition of osteolysis and increase of bone formation after local administration of siRNA-targeting RANK in a polyethylene particle-induced osteolysis model. Acta. Biomater. 13, 150–8 (2015). 25462844 10.1016/j.actbio.2014.10.042

[b29] Atkins G. J. *et al.* The induction of a catabolic phenotype in human primary osteoblasts and osteocytes by polyethylene particles. Biomaterials. 30, 3672–81 (2009). 19349075 10.1016/j.biomaterials.2009.03.035

[b30] Jiang Y., Jia T., Gong W., Wooley P. H. & Yang S. Y. Effects of Ti, PMMA, UHMWPE, and Co-Cr wear particles on differentiation and functions of bone marrow stromal cells. J. Biomed. Mater. Res. A. 101, 2817–25 (2013). 24039045 10.1002/jbm.a.34595 PMC3775288

[b31] He T. *et al.* Multiple biomarkers analysis for the early detection of prosthetic aseptic loosening of hip arthroplasty. Int. Orthop. 37, 1025–31 (2013). 23467893 10.1007/s00264-013-1837-1 PMC3664147

[b32] Ross R. D., Virdi A. S., Liu S., Sena K. & Sumner D. R. Particle-induced osteolysis is not accompanied by systemic remodeling but is reflected by systemic bone biomarkers. J. Orthop. Res. 32, 967–73 (2014). 24604767 10.1002/jor.22607

[b33] Lee S. S. *et al.* The effect of TNFalpha secreted from macrophages activated by titanium particles on osteogenic activity regulated by WNT/BMP signaling in osteoprogenitor cells. Biomaterials. 33, 4251–63 (2012). 22436801 10.1016/j.biomaterials.2012.03.005

[b34] Ren W. P. *et al.* Association between UHMWPE particle-induced inflammatory osteoclastogenesis and expression of RANKL, VEGF, and Flt-1 *in vivo*. Biomaterials. 27, 5161–9 (2006). 16814378 10.1016/j.biomaterials.2006.04.004

[b35] Wedemeyer C. *et al.* Particle-induced osteolysis in three-dimensional micro-computed tomography. Calcif. Tissue Int. 81, 394–402 (2007). 17952672 10.1007/s00223-007-9077-2

[b36] von Knoch M. *et al.* The effectiveness of polyethylene versus titanium particles in inducing osteolysis *in vivo*. J. Orthop. Res. 22, 237–43 (2004). 15013080 10.1016/j.orthres.2003.08.013

[b37] Geng D. *et al.* Protection against titanium particle induced osteolysis by cannabinoid receptor 2 selective antagonist. Biomaterials. 31, 1996–2000 (2010). 20004468 10.1016/j.biomaterials.2009.11.069

[b38] Livak K. J. & Schmittgen T.D. Analysis of relative gene expression data using real-time quantitative PCR and the 2(-Delta Delta C(T)) Method. Methods. 25, 402–8 (2001). 11846609 10.1006/meth.2001.1262

[b39] Kauther M. D. *et al.* RANKL-associated suppression of particle-induced osteolysis in an aged model of Calcitonin and alpha-CGRP deficiency. Biomaterials. 34, 2911–9 (2013). 23357366 10.1016/j.biomaterials.2013.01.034

[b40] Nich C. *et al.* Role of direct estrogen receptor signaling in wear particle-induced osteolysis. Biomaterials. 34, 641–50 (2013). 23113918 10.1016/j.biomaterials.2012.10.030 PMC4035303

[b41] Tian B. *et al.* The prevention of titanium-particle-induced osteolysis by OA-14 through the suppression of the p38 signaling pathway and inhibition of osteoclastogenesis. Biomaterials. 35, 8937–50 (2014). 25086794 10.1016/j.biomaterials.2014.06.055

